# Perspectives From Adolescents and Young Adults With Klinefelter Syndrome on Testosterone Supplementation and Fertility

**DOI:** 10.1210/jendso/bvaf112

**Published:** 2025-07-23

**Authors:** Abigail Tubman, Jaclyn L Papadakis, Courtney Finlayson, Debra Duquette, Allison Goetsch Weisman

**Affiliations:** Graduate Program in Genetic Counseling, Northwestern University, Chicago, IL 60611, USA; Department of Psychiatry & Behavioral Health, Ann & Robert H. Lurie Children's Hospital of Chicago, Chicago, IL 60611, USA; Department of Psychiatry & Behavioral Sciences, Northwestern University Feinberg School of Medicine, Chicago, IL 60611, USA; Department of Pediatrics, Northwestern University Feinberg School of Medicine, Chicago, IL 60611, USA; Division of Endocrinology, Ann and Robert H. Lurie Children's Hospital of Chicago, Chicago, IL 60611, USA; Graduate Program in Genetic Counseling, Northwestern University, Chicago, IL 60611, USA; Department of Medicine, Northwestern University Feinberg School of Medicine, Chicago, IL 60611, USA; Department of Pediatrics, Northwestern University Feinberg School of Medicine, Chicago, IL 60611, USA; Division of Endocrinology, Ann and Robert H. Lurie Children's Hospital of Chicago, Chicago, IL 60611, USA; Division of Genetics, Genomics, and Metabolism, Ann and Robert H. Lurie Children's Hospital of Chicago, Northwestern University Feinberg School of Medicine, Chicago, IL 60611, USA

**Keywords:** Klinefelter syndrome, infertility, testosterone therapy, treatment adherence, semen analysis, TESE

## Abstract

**Context:**

Infertility and testicular dysfunction are typical features of Klinefelter syndrome (KS; 47,XXY). KS affects ∼1:600 male births, making it the most common chromosomal aneuploidy. As more adolescent and young adult (AYA) patients with KS seek care, it becomes increasingly important to investigate their understanding of information about testosterone supplementation and infertility.

**Objective:**

This qualitative study aimed to explore the perspectives of AYA with KS about testicular dysfunction, specifically the likely need for testosterone replacement and, if desired, fertility-related interventions.

**Methods:**

Semistructured interviews were conducted with 13 AYA with KS ages 14 to 23 years (mean, age 16 years). Reflexive thematic analysis was applied.

**Results:**

Two themes were generated concerning testicular dysfunction: understanding of testosterone supplementation and fertility discussions. Most individuals lacked a comprehensive understanding of the use and health benefits of testosterone treatment. A facilitator to testosterone replacement adherence was perceived health benefits, whereas a barrier was the mode of delivery. All participants had either seen a fertility specialist or planned to see one. Emotional responses to infertility varied; however, many had negative reactions and emphasized the importance of learning that there are many ways to have children.

**Conclusion:**

This study improves the understanding of AYA with KS perspectives regarding testosterone supplementation and infertility, which in turn may help providers implement effective clinical and educational interventions for this growing patient population.

Klinefelter syndrome (KS) is characterized by a karyotype of 47,XXY and is the most common chromosomal aneuploidy, affecting approximately 1:600 male births [1, 2]. Due to a lack of distinct clinical presentation, many individuals remain undiagnosed or are diagnosed in adulthood subsequent to an infertility evaluation, as small testes, azoospermia, and hypergonadotropic hypogonadism are the most frequently seen characteristics among those with KS [1, 2].

The European Academy of Andrology released a guideline for the care of individuals with KS from childhood through adulthood in 2020, becoming the first and only professional society to do so [2]. Common medical concerns among those with KS including hormone replacement, infertility, and fertility preservation are addressed in this guideline. Testosterone supplementation is recommended in cases of delayed puberty and should be initiated in patients with KS with clinical hypogonadism [2, 3]. Additionally, most patients with KS (>90%) experience infertility because of nonobstructive azoospermia [1, 2, 4]. The guideline recommends that adolescents should be counselled on the impacts to fertility caused by KS and, if desired, undergo a fertility evaluation including a semen analysis and subsequent cryopreservation if sperm is present. Adolescents may also undergo specific counseling and a testicular biopsy for sperm extraction (TESE) with cryopreservation if sperm is not present in the semen analysis [2, 5]. Although this guideline addresses many facets of care for adolescents with KS, there are limited data on adolescent perspectives about testosterone replacement and the impact of KS on future fertility.

Previous research examining the impact of living with KS found that individuals with KS identified infertility as 1 of the greatest challenges faced [6, 7]. Specifically, adolescents and adults with KS recounted feelings of sadness and loss when faced with the potential for infertility [7]. Similarly, Papadakis et al (2021) observed that adolescents and young adults (AYA) with various differences of sex development (DSD; a group of diagnoses in which sex chromosomes, hormone levels, internal reproductive anatomy, and/or external genital appearance differ from typical binary male or female pathways of development [8]) found that the news of infertility had a mostly negative emotional impact on patients initially, but that this was not lasting because of individuals having a lack of interest in having children at that time in their lives [9].

It is becoming increasingly important to examine the understanding and perspectives of AYA with KS on aspects of their diagnosis such as testosterone supplementation and infertility due to the increase in size of this patient population. Previous estimates state that only ∼25% of individuals will receive a clinical diagnosis of KS, with a median age of diagnosis of 27 years [10, 11]. However, prenatal suspicion of X chromosome aneuploidy via cell free DNA screening technology has increased the overall diagnosis rates for individuals with KS and subsequently leads to a growing pediatric patient population [1].

Although there are recommendations for initiating testosterone therapy and fertility evaluation in AYA with KS, there is limited research on the perspectives and lived experience of AYA with KS about these integral aspects of the condition. The present study aimed to explore the experiences of AYA with KS regarding testicular dysfunction, specifically the likely need for testosterone replacement and fertility-related interventions, to help clinicians shape future conversations with AYA with KS on these topics.

## Material and Methods

### Study Design

The current study reports on data pertaining to the perspectives of AYA with KS about testicular dysfunction. Data for this study were collected by semistructured interviews and gathered as part of a broader qualitative study.

### Study Population and Recruitment

Eligible participants included individuals aged 14 to 25 years (minimum education level of ninth grade) with a diagnosis of KS (at least one 47,XXY cell line). A total of 54 eligible participants were identified via a previous retrospective chart review of 154 patients seen between 2012 and 2023 for KS at a single pediatric institution. Potential participants, or their guardians if younger than age 18 years, were contacted via MyChart (patient-accessible medical records database) message, email, and/or in person in the clinic with recruitment fliers, with a maximum of 3 attempted contacts. Participants were also recruited through Living with XXY, a US-based KS support organization, which advertised the study via newsletter mailings, website postings, and social media posts. Upon completion of all study procedures, participants received a $50 Visa gift card. The study was approved by the institutional review board of Ann & Robert H. Lurie Children's Hospital of Chicago (2023-6234). Informed consent was obtained from all participants older than age 18 years. Informed assent and parent/guardian consent were obtained for all participants younger than age 18 years.

### Data Collection

The authors collaboratively developed an interview guide (Supplement A 12) with open-ended questions exploring 3 domains: medical care needs including testosterone replacement and future fertility, utilization of support systems and resources, and psychosocial well-being. This guide was developed based primarily on the following: (1) an interview guide developed by Papadakis et al (2021) exploring fertility-related health care and decision-making needs for AYA with various DSDs; (2) literature review regarding individuals with KS and other DSDs [6, 7, 13, 14]; and (3) the authors’ expertise caring for children and AYA with KS (A.W.: genetic counselor; C.F.: endocrinologist; J.P.: pediatric psychologist).

Interviews took place between November 2023 and June 2024 and were conducted by 1 author (A.T.) online via Zoom (version 5.16.10) [15]. A.T. did not have a therapeutic relationship with any participants. The interviewer's video remained on for the duration of the interview, whereas participants had the option to leave their video off if desired. The interviewer conducted all interviews in a private space and participants were encouraged to do the same. The interviewer did not have a treatment relationship with any participants. All interviews were audio recorded, assigned a study ID, and transcribed by 1 author (A.T.) with identifying information, such as first names, places of employment, and names of schools removed. Sufficient informational power was achieved and data collection was subsequently concluded after 13 interviews. The research team determined that sufficient informational power had been reached based on the specificity of the participant population, in-depth interview data including strong quality of dialogue, and targeted aims of the study [16, 17].

### Data Analysis

A collaborative approach to coding was implemented which involved multiple meetings between authors in which they discussed the construction of meaning of codes. Authors A.T., J.P., and A.W. used the qualitative data analysis software, Dedoose Version 9.0.107 (2023), to analyze and code transcripts using an inductive and latent approach. The steps from Braun and Clarke [18-20] were used to generate themes from codes via reflexive thematic analysis [21]. This approach was used because it takes into account the positionality of the researchers in addition to biases in qualitative research. A social constructivist lens, which acknowledges that personal experiences create multiple realities [22], was used when analyzing the data. The codebook was developed inductively by A.T., J.P., and A.W.. Six transcripts were cross-coded by both A.T. and A.W., with A.T. applying the finalized codebook to all transcripts. The research team worked collaboratively through discussions to generate themes. Quotations were edited minimally for readability.

## Results

### Demographics

Thirteen male-identified individuals with KS participated. Seven of these participants were recruited through a single pediatric institution, whereas 6 participants were recruited through a support group. Participant demographics and interview characteristics are described in Table 1. The median length of interviews was 31 minutes (range, 17-44). Interviews for participants younger than age 18 years had a median length of 27 minutes (range, 17-41), whereas interviews for participants aged 18 years and older had a median length of 38 minutes (range, 27-44). All 13 participants identified their gender as male. Their median age at interview was 16 years (range, 14-23).

**Table 1.** Participant demographics

Characteristic	N	%
*Gender*		
Male	13	100
*Age at interview (y)*		
<18	7	54
18-25	6	46
Age at interview, y, median (range)		16 (14-23)
*Race/ethnicity*		
White	12	92
Asian (Pakistani)	1	8
*Level of education*		
In high school	7	54
In college	5	38
Completed college	1	8
**Recruitment source**		
Support group	6	46
Pediatric institution	7	54
**Length of interview**		
Age at interview (y)	Median length, minutes (range)	
<18	27 (17-41)	
18-25	38 (27-44)	
All interviews	31 (17-44)	
**Testosterone therapy use**		
*Active use*		
Total	6	46
Injection	5	83
Topical	1	17
*Previous use*		
Total	2	15
Injection	1	50
Oral	1	50
No use	5	38
**Fertility evaluation**		
Conversation about infertility with provider or parent	11	84
Visit with fertility specialist	6	46
Semen analysis	5	38
Testicular biopsy for sperm extraction	0	0

### Themes

**Figure 1.**
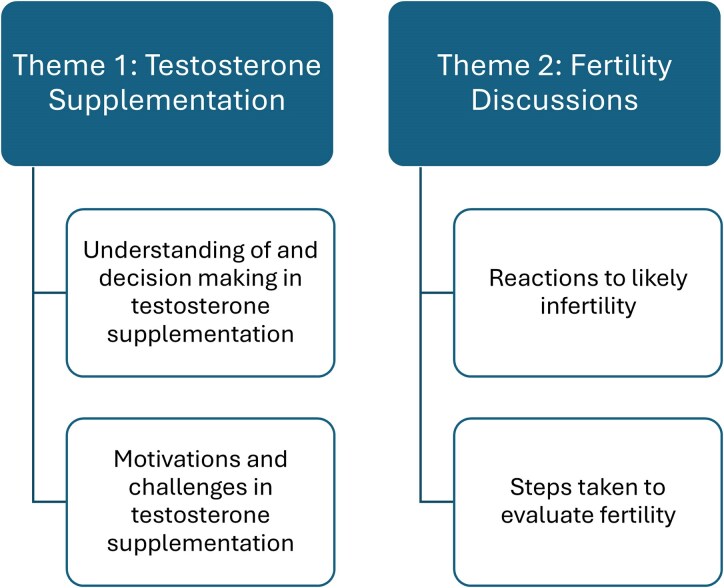
Summary of themes and subthemes.

After thematic analysis, 2 themes were generated regarding perceptions of AYA with KS toward testicular dysfunction, specifically the likely need for testosterone replacement and fertility related interventions: (1) testosterone supplementation and (2) fertility discussions. Figure 1 provides a summary of the themes and subthemes to be discussed.

### Testosterone Supplementation

**Figure 2.**
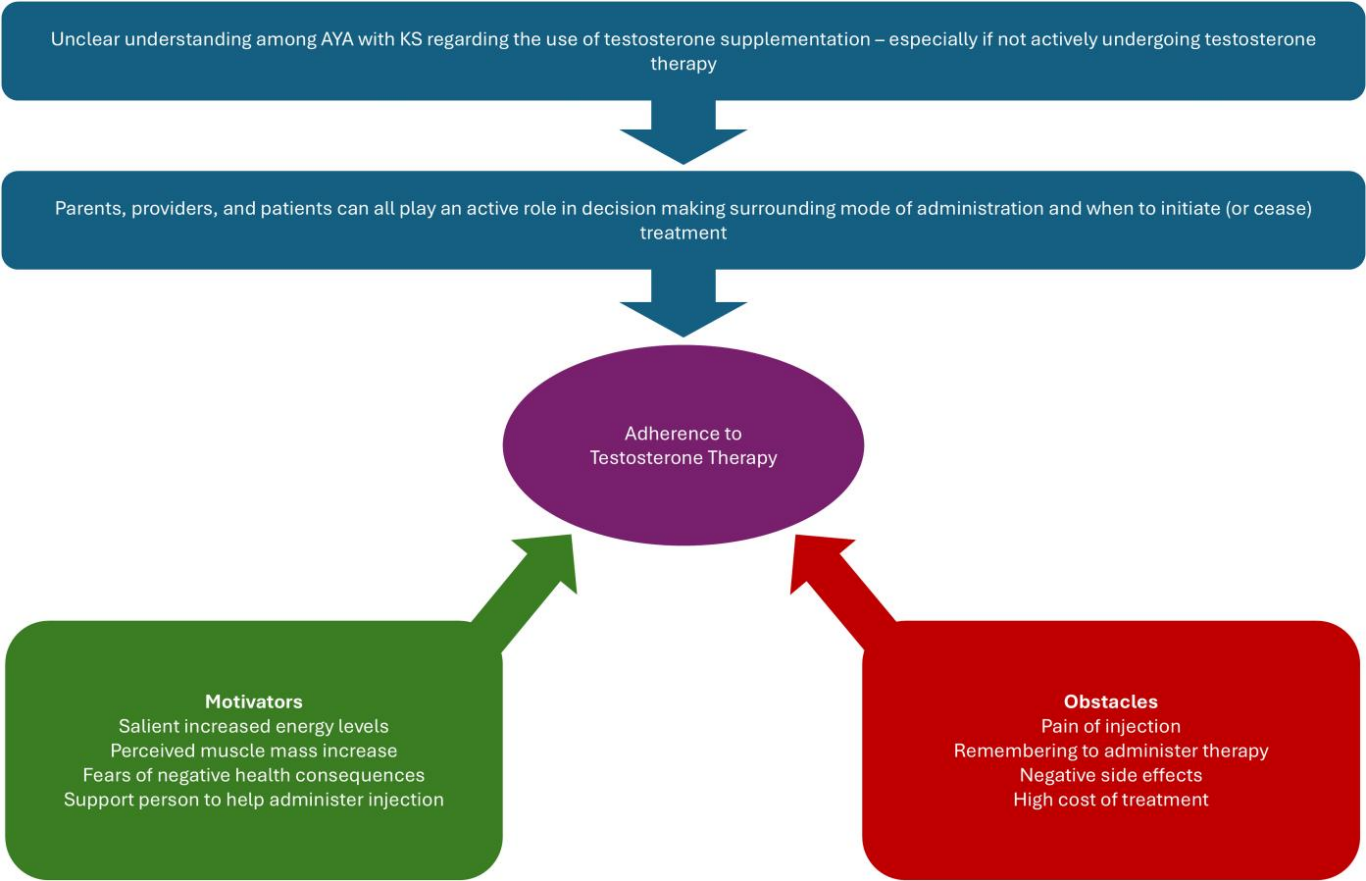
Summary of factors influencing adherence to testosterone therapy, including motivators and obstacles.

Two subthemes were created regarding testosterone supplementation: (1) understanding of and decision making in testosterone supplementation and (2) motivations and challenges in testosterone supplementation. Several factors were identified by participants as contributing to adherence to testosterone supplementation (Fig. 2).

Many participants were unclear on why people with KS may need testosterone supplementation or why they themselves were currently undergoing testosterone treatment, with the majority of participants echoing the following, “I don't have the best understanding. I think it's just cause when you have [KS] your testosterone levels aren't as high as a regular person” (ID#12, aged 16 years, no use of testosterone therapy). When asked to be more specific, the most common reasons cited were “the reason I’m taking it is to build up muscle” (ID#7, aged 19 years, active use of testosterone injections) and “I’ve heard it's supposed to help with working out” (ID#4, aged 20 years, previous use of testosterone injections). Two participants mentioned other health benefits including bone density (ID#11, aged 16 years, active use of testosterone injections) and initiating puberty (ID#10, aged 16 years, active use of testosterone injections). Overall, participants who were currently receiving testosterone therapy had a better understanding of the important role testosterone plays in the body and were able to more clearly verbalize that individuals with KS make less testosterone than individuals without KS. Additionally, of the 6 participants actively undergoing testosterone therapy, 5 were recruited through a support organization.

When asked about who was involved in decision making surrounding when to initiate, and for some when to cease, testosterone therapy, 5 participants identified themselves as playing an active role (ID#4, ID#5, ID#7, ID#9, ID#13), 6 identified their parents (ID#4, ID#7, ID#8 ID#10, ID#11, ID#13), and 8 identified their doctor (ID#1, ID#4, ID#5, ID#6, ID#7, ID#9, ID#11, ID#13). Some participants shed light on factors contributing to their decision regarding which type of testosterone treatment to administer. For example, 1 participant stated, “I felt like the lotion would be easiest […] I really don't like needles” (ID#13, aged 21 years, active use of testosterone gel), whereas another participant had the opposite thought stating, “[the shot is] just a one and done each week instead of I need to take a shower and put this cream on and make sure no one else in my family uses it” (ID#11, aged 16 years, active use of testosterone injections).

These quotations also illustrate some of the challenges that participants face when administering testosterone. By far the most cited challenge was the actual injection itself being painful; however, participants did note that the pain lessened over time, stating “at this point it's more of an annoyance” (ID#11, aged 16 years, active use of testosterone injections) and “I don't know how to explain it but you won't feel it anymore. Like your body will get used to that so it won't hurt as much” (ID#8, aged 14 years, active use of testosterone injections). Other challenges included problems remembering which day to administer the injection (ID#4, aged 20 years, previous use of testosterone injections; ID#10, aged 16 years, current use of testosterone injections), having anxiety leading up to the injection, “I always push it back cause I always get nervous still with the shot even though I know I’ll be fine. But it's just like the anxiety build up I guess” (ID#7, aged 19 years, current use of testosterone injections), and perceived negative side effects from the therapy including having a shorter temper (ID#1, aged 23 years, previous use of oral testosterone) and hot flashes (ID#10, aged 16 years, current use of testosterone injections). Three participants also mentioned insurance as a barrier due to lack of coverage, high costs associated with the medication, or administrative errors delaying treatment (ID#5, aged 21 years, no use of testosterone therapy; ID#10, aged 16 years, current use of testosterone injections; ID#13, aged 21 years, current use of testosterone gel).

Despite many participants describing challenges faced with administering testosterone, most described their motivation to comply with treatment as being linked to the benefits of therapy. The most common benefit of testosterone therapy was cited as having more energy (ID#1, ID#4, ID#9, ID#10, ID#11, ID#13), with 1 participant stating, “I just feel a lot more awake like I want to go outside more and I want to run around, I just feel a bit more alive” (ID#13, aged 21 years, current use of testosterone gel) and another saying “I have a boost of energy all the time” (ID#9, aged 15 years, current use of testosterone injections). Another positive effect was “I’ve noticed some changes, especially muscle […] it's easier to build muscle […] I feel more endurance too” (ID#7, aged 19 years, current use of testosterone injections). Some expressed fears over health consequences associated with noncompliance to testosterone therapy as a motivating factor (ID#10, aged 16 years, current use of testosterone injections; ID#13, aged 21 years, current use of testosterone gel), whereas others felt less inclined to continue treatment with 1 participant stating, “I’ve never noticed [testosterone] hav[ing] a significant difference. I guess that made it, here I am stabbing a needle into my leg every week for this small energy boost” (ID#4, aged 20 years, previous use of testosterone injections). One participant mentioned having a support person with you when administering the injection was helpful in decreasing anxiety (ID#7, aged 19 years, current use of testosterone injections). Additionally, reminders from parents about when to administer medication and check-ins about any negative or positive side effects participants may be experiencing were helpful in increasing compliance among 2 participants (ID#10, aged 16 years, current use of testosterone injections; ID#11, aged 16 years, current use of testosterone injections).

### Fertility Discussions

Two subthemes were created regarding fertility discussions: (1) reactions to likely infertility and (2) steps taken to evaluate fertility.

Of the 13 participants, 11 had discussed fertility either with a doctor or their parents. The 2 participants who had not discussed fertility (ID#3 and ID#9) were 2 of the younger participants (both being 15 years old at the time of the interview). Participants largely felt that these were difficult conversations that were uncomfortable at times, with 1 participant stating, “I think that's the hardest thing to hear as a young adult because you don't know what you really want in life […] it's the hardest thing to hear that you probably won't have kids” (ID#5, aged 15 years). The feeling of loss associated with infertility coupled with a young age where participants were unsure of future family building plans was shared among others with 1 participant saying, “for being that young, and I can't say this for everybody, but I would say most people want kids, so I broke down and started to cry” (ID#2, aged 21 years). However, not every participant experienced a negative reaction when learning about their high chance for infertility, with 1 participant saying, “to be honest I’m fine with not being able to have kids […] they’re just loud and annoying […] I don't really have any negative emotions about talking about it” (ID#11, aged 16 years).

Many participants mentioned alternative methods for having children and expressed hope for building a family in the future despite not knowing exactly what that looks like currently, with talk of sperm donors (ID#9, aged 15 years), future medical advances (ID#12, aged 16 years), and adoption (ID#9, aged 15 years; ID#10, aged 16 years; ID#4, aged 20 years). Participants found it helpful to discuss these alternative options for having children with 1 participant saying, “my parents were reassuring me that we’ll find a way” (ID#2, aged 21 years) and another stating, “I was talking to my best friend about how I may never be able to have kids and he was like ‘there's a lot of other options and routes you can take to fulfill that’ and that did make me feel a little better” (ID#1, aged 23 years).

Participants implemented various strategies to cope with negative feelings surrounding infertility including religion (“[my parents] say ‘don't think about it’ since we’re religious and Muslim they’re just like ‘let God take it, leave it in God's hands’ (ID#2, aged 21 years)) and humor (“at this point I kind of joke about it like ‘haha couldn't be my kid’” (ID#4, aged 20 years)). None of the participants were actively family planning, mostly due to their young age at the time of the interview, leading to some avoidance, for example, “I just didn't think about it after cause it's not in my immediate future for at least another what like 10 years or something” (ID#7, aged 19 years) and “I want to have my own kids but it's just something in the future I’ll have to worry about but I’m not focused on it right now, I’m 16” (ID#12, aged 16 years).

Seeking additional information about fertility status and options for future family building was discussed by many participants as being helpful in coping with some of the negative feelings they were experiencing. All of the participants either had already seen a fertility specialist or expressed that they plan to see one in the future. Many expressed that they viewed the fertility specialist as a place to get more information and urge others with KS to use them as a resource stating, “don't be nervous […] they really do know what's best so just don't be nervous, have an open mind, and take it one step at a time” (ID#1, aged 23 years).

**Figure 3.**
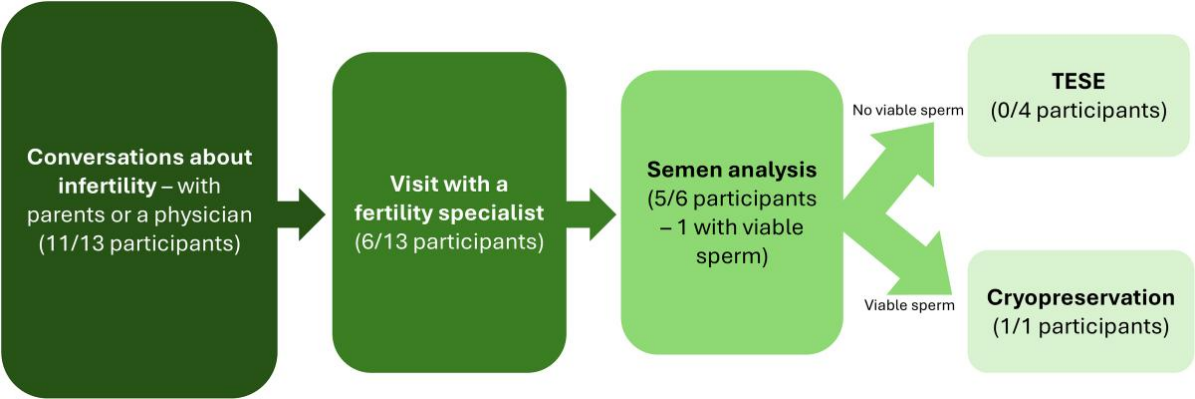
Steps taken by participants to evaluate fertility.

Of the 6 participants who have already met with a fertility specialist, 5 had undergone at least 1 semen analysis (ID#1, aged 23 years; ID#4, aged 20 years; ID#5, aged 21 years; ID#7, aged 19 years; ID#13, aged 21 years), and of those 5, 1 had viable sperm in his sample. The participant with viable sperm decided to pursue cryopreservation to have “an option for later in life” (ID#13, aged 21 years). The main motivation for providing a semen sample was not wanting to have regrets, with 1 participant stating, “I don't want to have regrets in life, I don't want to be 50 years old going ‘I really should have done that’ but now it's too late” (ID#5, aged 21 years). Five participants had been informed about the TESE procedure (ID#4, aged 20 years; ID#5, aged 21 years; ID#7, aged 19 years; ID#10, aged 16 years; ID#13, aged 21 years); however, no participants had undergone a TESE procedure at the time of the interview. In fact, many were reluctant to pursue this option with participants saying, “that did not sound pleasant to me so I said no thank you” (ID#7, aged 19 years) and “I was told to have another test where they essentially stick a needle into my testicles which I said no thank you to cause I would rather be infertile than deal with that” (ID#4; aged 20 years). One participant did express that he would consider this option if his future partner desired biological children (ID#5, aged 21 years). A summary of the steps taken by participants to understand or evaluate fertility is shown in Fig. 3.

## Discussion

The current study is the first to our knowledge to use qualitative methods to explore the perspectives of AYA with KS regarding testicular dysfunction. AYA with KS shared their understanding of testosterone therapy in addition to what benefits and challenges they perceive to be associated with this treatment. Additionally, AYA with KS shared their perceptions of the likely infertility associated with their diagnosis and detailed their journey in seeking additional information for future family planning. We report several themes that can inform current clinical management, specifically regarding counseling AYA with KS in understanding the testicular dysfunction aspect of their diagnosis.

Participants in this study identified several barriers to adhering to testosterone treatment including pain from the injection itself, forgetting to administer therapy, perceived negative side effects from treatment, and high costs of medication or delays because of obtaining insurance coverage. This is in contrast to a previous study of mostly adults (average age, 40.7 years) with KS, which found that only 9% of their participants had challenges with testosterone treatment with the main concerns being the associated cost and mitigating negative side effects [7]. This difference could be explained by the young age of participants in the present study accompanied with the fact that many of them had either started testosterone more recently than the adults in the aforementioned study or were not on treatment at all. Although research is limited in adherence to testosterone therapy in AYA with KS, a study on adherence to treatment for AYA with various other chronic illnesses showed that age, poor psychological adjustment, unfamiliarity with the health care system, and financial barriers were all correlated to nonadherence to treatment [23]. One factor identified by participants in the present study that promoted adherence to testosterone therapy was perceived benefits that individuals experienced as a result of this therapy. This finding can inform clinicians’ approach to educating patients on the importance of testosterone therapy as the vast majority of participants in the present study did not fully comprehend why they were being prescribed testosterone. Additionally, clinicians should regularly assess for barriers to adherence in order to address these concerns for patients.

While there are many important medical benefits to testosterone therapy in patients with KS [2], the most cited positive impacts by participants in this study were increases in muscle mass and energy levels. It's unclear if these benefits were emphasized by clinicians or this is what these participants took away from medical conversations because of its personal relevance to them. Previous studies on testosterone supplementation and quality of life in men with KS have yielded conflicting results with the majority, suggesting that supplementation with testosterone does not increase quality of life for these individuals [11, 24, 25]. Despite this, results of this study and others indicate that it may be important for specialty care providers to highlight benefits that AYA with KS may experience as a result of testosterone therapy to increase adherence and result in reduced disease morbidity [14].

Another important aspect to medical management for AYA with KS is providing counseling for infertility and, if desired, performing a fertility evaluation [2]. Currently, there is not a clear consensus on when and how to have fertility-related discussions with adolescents with KS, with providers having different practices for fertility evaluations [26, 27]. Previous recommendations for fertility counseling for youth at risk for fertility impairment suggest that conversations should begin early and be ongoing [28]. In the present study, all but 2 participants had discussed fertility to some extent, with many finding initial conversations about fertility to be difficult and emotional at times. Prior research has shown that men with KS report that infertility is the greatest challenge they face as part of their condition [7]. Although some participants in this study did describe feelings of sorrow and loss when initially learning about their infertility, this was not an experience shared by all participants, with many expressing that it's something they will face in the future but have hope for family building. This is consistent with prior research in AYA with various DSDs, excluding KS, which found that despite negative initial emotional reactions, participants came to accept their fertility status and were optimistic about fertility and parenting options that may be available in the future [9].

Conversations surrounding fertility evaluation and preservation should be ongoing and tailored to the patient's maturity [29, 30]. Participants in the present study placed a high value in learning more about how their fertility is impacted by KS through conversations with a fertility specialist and fertility evaluation with the primary motivating factor being not wanting to have regrets later in life. This is consistent with previous research, which showed that individuals with DSDs valued learning about fertility impairment at younger ages so they were able to better cope with and integrate the information [9, 31-33].

Fertility evaluations can involve an individual giving a semen sample for analysis of viable spermatozoa, which if found, can be cryopreserved for later use. Approximately 90% of individuals with KS are azoospermic, meaning there is no viable spermatozoa in their ejaculate [2]. In the present study, 4 of the 5 participants who had performed a semen analysis reported azoospermia (80%). Individuals, including adolescents, with KS who are found to be azoospermic may be offered a TESE [2]. The likelihood of success in finding sperm via TESE seem to be independent of age between 14 and 23 years old, suggesting that this procedure could be deferred to adulthood for young men with KS [30]. Although participants in the present study had been informed and offered TESE, none had pursued this option of fertility preservation. This is likely because of the young age of participants in addition to none of them actively family planning. The decision of whether or not to undergo a TESE is multifaceted and individual. In a study on the surgical experiences of youth with DSDs, excluding KS, participants found that regular and ongoing communication with providers on a multidisciplinary team before and after surgery was helpful in the decision-making process [32].

The findings in the present study along with their practice implications are summarized in Table 2.

**Table 2.** Clinical considerations for providers caring for AYA patients with KS

Study finding	Clinical consideration
Theme 1: Testosterone treatment	Emphasize the physical benefits associated with testosterone therapy to increase treatment motivation and compliance. It may be helpful for some patients to focus on more salient benefits to treatment such as increases in muscle mass and energy levels as mentioned by participants in this study. For other patients, it may be more helpful to focus on important long-term health benefits such as cardiovascular health and bone density.
Theme 2: Fertility discussions	Highlight that there are many ways to have children and ensure education about all potential family-building options, including use of donor sperm and adoption.
	Encourage AYA with KS to speak to a fertility specialist as a way to gain deeper understanding of their fertility potential, as well as fertility preservation and alternative family building options.

Abbreviations: AYA, adolescent and young adult; KS, Klinefelter syndrome.

The perspectives outlined in this study are not representative of all AYA with KS. Most participants in the present study identified as White; therefore, there is a lack of racial and ethnic diversity. Future research should focus on including people of color with KS in addition to people with various cultural backgrounds as their perspectives may differ from those represented in this study. Additionally, because all participants identified as male, future research exploring how individuals with KS with other gender identities feel about the topics discussed is needed. Recruitment through a support group is associated with limitations in that these participants have sought out additional support and information regarding KS. Additionally, recruitment via a multidisciplinary clinic at a single large academic pediatric medical center located in an urban area introduced potential biases as this represents 1 institution's patient population. Recruitment materials also included that questions on testosterone therapy and fertility preservation would be asked during the interview. This could have deterred individuals who were less familiar with or less comfortable discussing those topics from choosing to participate. Methods such as member checking or triangulation were not used during this analysis. This underscores the importance of future studies to build upon the findings of this study and to validate the perspectives shared by these participants.

## Conclusions

This study was the first to our knowledge to qualitatively explore AYA perspectives on the impact of testicular dysfunction in KS, including testosterone supplementation and fertility impairment. Results revealed potential areas for improvement in helping AYA with KS understand the benefits of testosterone therapy to increase their motivation for treatment adherence. Additionally, this study revealed that AYA with KS benefited from meeting with a fertility specialist as a way to get more information about their own fertility potential and what future parenting options could be available to them. Results of this study aim to help provide a foundation for future research and educational and clinical interventions to better support this growing patient population in adolescence and young adulthood.

## Data Availability

Restrictions apply to the availability of some or all data generated or analyzed during this study to preserve patient confidentiality or because they were used under license. The corresponding author will on request detail the restrictions and any conditions under which access to some data may be provided.

## Abbreviations

AYA: adolescent and young adult
DSD: differences of sex development
KS: Klinefelter syndrome
TESE: testicular biopsy for sperm extraction
